# Reoperations for Bleeding Portal Hypertension. Surgical Rescue of Surgical Failures

**DOI:** 10.1155/1999/73414

**Published:** 1999-04

**Authors:** Miguel Angel Mercado, Tito José María Gómez-Méndez, Julio César Morales-Linares, Jorge Granados, Carlos Chan, Gilberto Rojas, Héctor Orozco

**Affiliations:** Portal Hypertension Clinic Instituto Nacional de la Nutrición Salvador Zubirán Vasco de Quiroga 15 México, D.F. Tlalpan 14000 Mexico

## Abstract

*Background;*  Surgery for portal hypertension has a low rebleeding rate. Patients that rebleed can be grossly divided into those who die as a consequence of the episode, those who don't die but develop liver failure (remaining as Child-Pugh C) and those who, in spite of the bleeding episode, retain good liver function (Child-Pugh A or B). At our hospital, the latter group is considered for further surgical treatment. We report here the results of surgical rescue of surgical failures.

*Methods;* In a twenty year period, 36 patients (30 Child-Pugh A, 6 Child-Pugh B) were reoperated. The files of these patients were reviewed.

*Results;* Average age was 33 years. Cirrhosis was present in 31 cases. All patients were electively reoperated with portal blood flow preserving procedures. Operative mortality for the whole group was 12% and for the Child-Pugh A group 6.6%. Rebleeding was observed in 5.5%. Postoperative incapacitating encephalopathy was recorded in one case (2.7%). Good quality of life was recorded in 84% of the cases. Survival (Kaplan-Meier) was 78% at 6 months and 69% at 5 years.

*Conclusions;* Surgical failures in low risk patients (Child-Pugh A or B) can be treated by means of surgery, and a low mortality, re-bleeding and encephalopathy rate can be expected. The performance of a portal blood flow preserving procedure is recommended.


HPB Surgery, 1999, Vol. 11, pp. 151-155
Reprints available directly from the publisher
Photocopying permitted by license only

© 1999 OPA (Overseas Publishers Association) N.V.
Published by license under
the Harwood Academic Publishers imprint,
part of The Gordon and Breach Publishing Group.
Printed in Malaysia.

### Reoperations for Bleeding Portal Hypertension. Surgical Rescue of Surgical Failures

MIGUEL ANGEL MERCADO*, TITO JOSÉ MARÍA GÓMEZ-MÉNDEZ, JULIO CÉSAR MORALES-LINARES, JORGE GRANADOS, CARLOS CHAN, GILBERTO ROJAS and HÉCTOR OROZCO

*Portal Hypertension Clinic, Instituto Nacional de la Nutrición, Salvador Zubirán, Vasco de Quiroga 15, Tlalpan 14000, México, D.F., Mexico*

*(Received 25 June 1997; In final form 10 December 1997)*

***Background;*** **Surgery for portal hypertension has a low rebleeding rate. Patients that rebleed can be grossly divided into those who die as a consequence of the episode, those who don't die but develop liver failure (remaining as Child-Pugh C) and those who, in spite of the bleeding episode, retain good liver function (Child-Pugh A or B). At our hospital, the latter group is considered for further surgical treatment. We report here the results of surgical rescue of surgical failures.**

***Methods;*** **In a twenty year period, 36 patients (30 Child-Pugh A, 6 Child-Pugh B) were reoperated. The files of these patients were reviewed.**

***Results;*** **Average age was 33 years. Cirrhosis was present in 31 cases. All patients were electively reoperated with portal blood flow preserving procedures. Operative mortality for the whole group was 12% and for the Child-Pugh A group 6.6%. Rebleeding was observed in 5.5%. Postoperative incapacitating encephalopathy was recorded in one case (2.7%). Good quality of life was recorded in 84% of the cases. Survival (Kaplan-Meier) was 78% at 6 months and 69% at 5 years.**

***Conclusions;*** **Surgical failures in low risk patients (Child-Pugh A or B) can be treated by means of surgery, and a low mortality, re-bleeding and encephalopathy rate can be expected. The performance of a portal blood flow preserving procedure is recommended.**

*Keywords:* Portal hypertension surgery, reoperations

*Corresponding author. Tel.: 573-9321; Fax: 655-1076.

The most effective treatment for hemorrhagic portal hypertension (HPH) is surgery. It has the lowest re-bleeding rate among the different treatment alternatives. The types of procedures and their indications have evolved in the last decade [1]. It has been demonstrated that the main indication for surgery is in patients with a history of bleeding and good liver function (low risk patients) and these patients should be operated on in an elective fashion [2]. Portal blood flow preserving procedures (selective shunts, Sugiura-Futagawa procedure, low diameter shunts) are the preferred operation for most patients. In many centers, a low operative mortality as well as a low re-bleeding and encephalopathy rate has been achieved. There is no doubt that for low risk patients, portal blood flow preserving procedures are the treatment of choice [3].

Re-bleeding rates after surgery are usually below 10% [4-7]. Patients who rebled can be grossly divided into those who die as a consequence of the episode, those who develop liver failure and those who spite of the bleeding episode, recover and remain with good liver function (Child-Pugh A or B).

Many centers manage surgical failures with other forms of therapy which include sclerotherapy, intrahepatic shunts (TIPS) and in some instances with liver transplants. Sclerotherapy has a high re-bleeding rate (30% to 50%) while TIPS have a high rate of dysfunction. Liver transplants are reserved for those patients with bad liver function.

Here, we report our results with the surgical management of previously operated HPH patients with good liver function, that is, the surgical rescue of surgical failures.

### METHODS

In the last two decades, surgical treatment of portal hypertension has been standardized in our hospital. No prophylactic cases are operated. Only patients with a good liver function are electively operated using portal blood flow preserving procedures. These are the selective shunts, the Sugiura-Futagawa operation and in the last 5 years, also low diameter shunts [3].

The patients selected for surgery must fulfill the following criteria [5]:

(a) History of bleeding secondary to portal hypertension.
(b) Good cardiopulmonary and renal function
(c) Good liver function

- Albumin >3.5 gr/dl
- Total bilirubin <2 mg/dl
- Prothrombin time <2 seconds
- No encephalopathy
- No ascitis

(d) Good nutritional status

All patients selected for surgery, including those with a history of surgery, are subjected to an angiographic evaluation of the arterial and venous phases of the celiac trunk and mesenteric artery. According to the anatomical findings and characteristics of the splenomesenteric portal system, patients are selected to receive a selective shunt (distal splenorenal or splenocaval), a Sugiura-Futagawa operation, or a low diameter (mesocaval) shunt. After the operation, routine follow-up is performed and in the cases with a shunt operation, angiograms are done to evaluate the shunt patency.

The files of the patients reoperated on for re-bleeding were reviewed. Demographic data of the patients were recorded as well as the results of the reoperation. Early and late mortality, postoperative encephalopathy, re-bleeding, survival and quality of life were studied [8].

### RESULTS

In a 20 year period, among 667 patients operated for bleeding portal hypertension, 36 were found to have a reoperation. Patients that had bleeding from other sources (not related to portal hypertension) were excluded. There were 23 male and 13 female patients. Age average was 33 years (range 14 to 66 years). In 31 cases, hepatic cirrhosis was demonstrated. Two had congenital hepatic fibrosis an three portal vein thrombosis. All patients had a liver biopsy at the operation. According to the Child-Pugh classification, 30 patients were classified as A (71%) and six (14%) as Child-Pugh B. All patients were electively reoperated and four patients died in the first 30 postoperative days and recorded as operative mortality (12%). According to the Child Pugh classification, 2 cases corresponded to the group A (6.6%) (Tab. I). At the time of review, 23 patients were alive and well. Seven late mortalities were recorded and two cases were lost to follow up. The main cause of death was liver failure.

In Table II, the information about the failure and cause of rebleeding and the kind of the reoperation is given. Thirty cases were failures from our hospital and six cases came from other hospitals. In all instances, the reoperations

TABLE I Mortality

| Early (0–30 days) | | Late (>30 days) | | |
|---|---|---|---|---|
| Child | Cause | Child | Cause | Time |
| A | Liver failure | A | HIV infection | 45 months |
| A | Multiorganic failure | A | Liver failure | 3 " |
| B | Liver failure | A | Liver failure | 6 " |
| B | Multiorganic failure | B | Re-bleeding | 7 " |
| | | A | Liver failure | 52 " |
| | | B | Liver failure | 2 " |
| | | A | Re-bleeding | 4 " |

TABLE II Previous operations and reoperations

| Previous operation | | Rebleeding | | Reoperation | |
|---|---|---|---|---|---|
| Devascularization | 12 | Esophagogastric Varices | 8 | Sugiura-Futagawa | 10 |
| | | Gastric Varices | 4 | Selective shunt | 1 |
| | | Gastropathy | 0 | Mesocaval | 1 |
| Sugiura-Futagawa | 9 | Esophagogastric Varices | 3 | Low diameter mesocaval shunt | 8 |
| Complete | 6 | | | Thoracic stage of SF | 1** |
| Abdominal | 3 | Gastric Varices (Fundic) | 4 | | |
| | | Gastropathy | 2 | | |
| Selective shunt | 12* | Esophagogastric Varices | 12 | Sugiura-Futagawa | 6 |
| DSRS | 5 | | | Selective shunt | 3** |
| Splenocaval | 6 | | | Devascularizations | 1 |
| Coronario caval | 1 | | | Mesocaval | 1 |
| Total shunts | 2* | Esophagogastric Varices | 2 | Sugiura-Futagawa | 2 |
| Mesocaval | 1 | | | | |
| Portocaval | 1 | | | | |

* Obstructed shunts.
** A DSRS was converted to splenocaval shunt. Two splenocaval were converted to a DSRS.

performed were portal blood flow preserving procedures. Operative interval was between 1 and 5 years for 9 patients, between 5 and 10 years for 9 patients and more than 10 years for five patients.

Re-bleeding was recorded in two of the 36 cases (5.5%) with a mean follow up to 60 months. The two patients died in the 4th and 7th postoperative months. Postoperative encephalopathy was recorded in one case (2.7%) after reoperation. This patient had history of Sugiura-Futagawa operation that failed in the first year and a low diameter mesocaval shunt (10 mm ringed PTFE) was done. According to our criteria [8] for evaluation of quality of life, it was good for 84% of the cases and bad for 16% of cases. Survival (Kaplan-Meier) was 78% at 6 months and 69% at 5 years. (Fig. 1).

### DISCUSSION

Among the alternatives to control variceal bleeding, surgery remains as the most effective treatment. Re-bleeding rate is low in most series. Portal blood flow preserving procedures are the preferred operations [3]. In almost all centers in which surgery is routinely performed for the treatment of bleeding portal hypertension, selection of cases has been over emphasized. If surgery is electively performed in low risk patients (Child-Pugh A), good results are to be expected. Most of these patients do well after the operation, with a good quality of life [8].

Nevertheless, surgery has failures and there is virtually no series in which re-bleeding is not reported. For shunt surgery, the most likely cause of re-bleeding is shunt dysfunction.

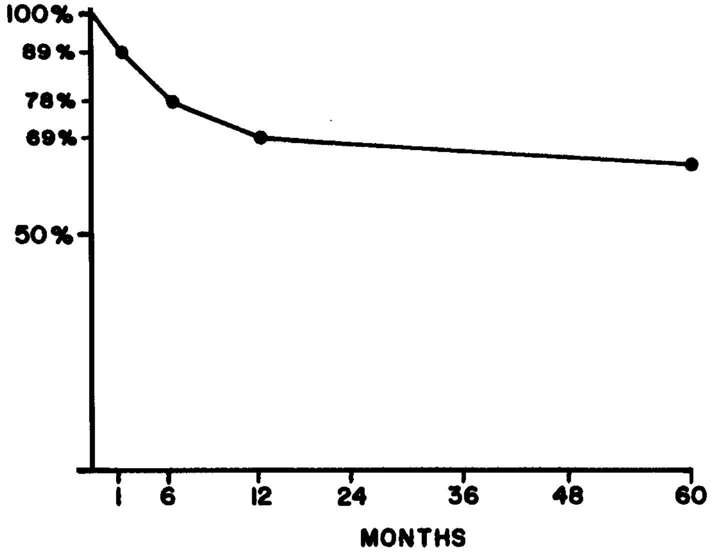

FIGURE 1 Survival curve for our series of 36 patients.

Selective shunts have a very low rate of obstruction and/or dysfunction [9, 10], less than 5% in our experience. For devascularizations, the cause is more difficult to explain, but certainly in spite of interrupting afferent and afferent vessels, portal hypertension still remains in the esophagogastric area, and variceal recurrence may occur. This event is less frequent in our experience for the extensive Sugiura-Futagawa devascularization.

Patients that fail to surgery have the possibility of rebleeding from other sources. This paper discusses only the patients that had rebleeding because of portal hypertension.

For patients with obstructed shunts esophagogastric varices is the most common site of rebleeding. This is particularly true for the DSRS, in which the whole esophagogastric and splenic area is drained through the shunt. For patients with devascularization procedures, gastric varices and hypertensive gastropathy represent a considerable source of bleeding.

In some cases, re-bleeding has a fatal outcome. In other cases, liver function deteriorates after re-bleeding. In another subset of patients, no changes in liver function are observed and they remain as Child-Pugh A. There is no agreement on how to treat these patients. In many centers, surgical failures are treated with alternate therapeutic choices, including sclerotherapy, TIPS and liver transplantation. Sclerotherapy has a high re-bleeding rate and in our opinion should be reserved for patients with bad liver function [2]. Liver transplantation is not indicated as treatment for re-bleeding, but there is no doubt that for patients with bad liver function, it is the treatment of choice [12], and TIPS are indicated as "a bridge" for liver transplantation and its role for long term treatment has not established yet. The high rate of dysfunction (as well as the fact that it is a non portal blood flow preserving procedure) does not make TIPS the treatment of choice for patients with good liver function [13].

The results reported here support the surgical rescue for surgical failures, for those patients with good liver function. Although reoperations are technically more difficult because of adhesions due to the previous procedure which makes intraoperative blood loss significantly higher. The operative mortality is low for patients with good liver function (less than

10% for Child A cases). Results are similar for the low risk patients operated for the first time. There is a low re-bleeding rate after the operation (less than 5%) and if a portal blood flow preserving procedure is done, a low encephalopathy rate is obtained.

The kind of procedure to be done depends on many variables. Some of these patients have no spleen or have an inadequate splenic vein. So, if it is not possible to do a selective shunt, these patients should be considered for a Sugiura-Futagawa procedure or a low diameter shunt (if the portal and mesenteric veins are adequate).

Our preference for the cases in which a selective shunt is not possible is to perform a Sugiura-Futagawa operation. Our results with low diameter shunts, although encouraging, are not as good a those obtained with the selective shunts and the Sugiura-Futagawa procedure. So, we reserve low diameter shunts for the cases in which a selective shunt or the Sugiura-Futagawa procedure is not possible, for example, a patient with history of esophagogastric devascularization and absence of spleen or inadequate spleen vein.

In some cases with a history of devascularization and an inadequate spleno meso portal system, a Sugiura-Futagawa operation has to be done. For these cases with a history of abdominal devascularization, a trans thoracic approach to perform the devascularization and esophageal transection has to be done.

It is concluded that surgical failures have to be treated by means of surgery, if the patient has good liver function after re-bleeding and can be electively operated. We strongly recommend the performance of a portal blood flow preserving procedures. We advice that shunt failures should be treated with the Sugiura-Futagawa operation, considering low diameter shunts as another alternative. Failure of devascularizations can be treated with shunts and in certain selected circumstances (no adequate vessels) devascularizations have to be converted to the Sugiura-Futagawa operation.

### *References*

[1] Rikkers, L. F., Sorrell, W. T. and Jin, G. (1992). Which portosystemic shunt is best?, *Gastroenterol. Clin. North. Am.*, **21**, 179–96.

[2] Orozco, H. and Mercado, M. A. (1994). Surgery for portal hypertension in 1994. In: Bosch J. and Groszman R. J. (Ed.). *Portal hypertension Pathophysiology and treatment*, Oxford Blackwell, pp. 180–85.

[3] Mercado, M. A., Takahashi, T., Rojas, G., Prado, E., Hernández, J., Tielve, M. and Orozco, H. (1993). Surgery in portal hypertension. Which patient and which operation? *Rev. Invest. Clin.*, **45**, 329–37.

[4] Warren, W. D., Millikan, W. J., Henderson, J. M. *et al.* (1982). Ten years portal hypertensive surgery at Emory, *Ann. Surg.*, **195**, 530–42.

[5] Paquet, K. J., Mercado, M. A., Koussouris, P., Kalk, J. F., Siemens, F., Cuan-Orozco, F. (1989). Improved results with selective distal splenorenal shunt in a highly selected patient population, *Ann. Surg.*, **210**, 184–89.

[6] Rypins, E. B., Sarfeh, I. J. (1990). Small diameter portocaval H-graft for variceal hemorrhage, *Surg. Clin. North. Am.*, **70**, 395–404.

[7] Sugiura, M., Futagawa, S. (1977). Further evaluation of the Sugiura procedure on the treatment of esophageal varices, *Arch. Surg.*, **112**, 1317–20.

[8] Orozco, H., Mercado, M. A., Takahashi, T., Rojas, G., Hernández, J., Tielve, M. (1994). Survival and quality of life after portal blood flow preserving procedures in patients with portal hypertension and liver cirrhosis, *Am. J. Surg.*, **168**, 10–15.

[9] Orozco, H., Mercado, M. A., Takahashi, T., García-Tsao, G., Guevara, L., Hernández, J. *et al.* (1990). Role of the distal splenorenal shunt in the management of variceal bleeding in Latin America, *Am. J. Surg.*, **160**, 86–9.

[10] Henderson, J. M. (1990). The distal splenorenal shunt, *Surg. Clin. North. Am.*, **70**, 405–23.

[11] Wexler, M. J., Stein, B. L. (1990). Non shunting operations for variceal hemorrhage, *Surg. Clin. North. Am.*, **70**, 425–48.

[12] Henderson, J. M. (1992). Liver transplantation for portal hypertension, *Gastroenterol. Clin. North. Am.*, **21**, 197–213.

[13] Burroughs, A. K., McCormick, P. A. (1994). TIPS: here the initial expectations been fulfillled?. In: Bosch J. Groszman R. J. (Eds.). Portal hypertension pathophysiology and treatment. Oxford Blackwell, pp. 164–79.

